# Prospects for using charity lotteries in social marketing

**DOI:** 10.12688/f1000research.114652.1

**Published:** 2022-06-16

**Authors:** Sergey Evgenievich Barykin, Svetlana Bozhuk, Nelli Kozlova, Nataliia Krasnostavskaia, Reena Mehta, Stepan Vinokurov, Inga Nimenia, Irina Vasilievna Kapustina, Elena Naumova, Natalia Dedyukhina

**Affiliations:** 1 Graduate School of Service and Trade, Peter the Great St. Petersburg Polytechnic University, St. Petersburg, Russian Federation; 2 K.J. Somaiya Institute of Management, Mumbai, India; 3 Department of economics, St Petersburg State University of Economics (UNECON), St Petersburg, Russian Federation; 4 Branch office Management Company, LOMO-Estate, Saint Petersburg, Russian Federation; 5 Saint Petersburg State Maritime Technical University, Saint Petersburg, Russian Federation; 6 Emperor Alexander, St. Petersburg State Transport University, St. Petersburg, Russian Federation

**Keywords:** social marketing, CSR, charity lotteries, consumer behavior, theory of planned behavior

## Abstract

**Background: **The purpose of this work is to study the prospects of using charity lotteries as a marketing tool for involving consumers and employees in charitable activities.

**Methods:** The research methodology is based on the principles of the theory of planned behavior by Ajzen.

**Results:** The study confirmed that behavioral intention is correlated with actual behavior. Subjective norms factors have the most significant influence on behavioral intention.

**Conclusions:** Correlation analysis allowed us to establish a weak effect of socio-demographic characteristics (Age, Gender, Capital Status, Ownership of Home, Educational Qualification, Employment, Annual Income) on behavior. The experience of participating in the lottery in the past also turned out to be insignificant. There is reason to believe that Russian consumers' decision to participate in the charity lottery is impulsive or due to pressure from their surroundings.

## Introduction

In Russia, charity as a type of social activity of the population has existed for a long time, but charity lotteries are rarely used.

Charitable organizations, whose purpose is to accumulate funds for gratuitous assistance, cannot have the funds necessary for the prize fund due to legislative regulation. Thus, charity lotteries in Russia can be commercial organizations that carry out long-term cooperation with a charitable foundation.

Practice shows that a commercial organization uses charitable events within the corporate social responsibility (CSR) policy framework to create new value for the consumer. Companies can also involve their employees in charity events, which affects the morale of the team. Thus, holding a charity lottery serves the goals of both the internal and external strategy of the organization.

Volunteer movement (providing action assistance), charitable fundraising (financial aid), and charity lotteries are usually considered options for involving people in philanthropic projects. Compared with other ways of attracting people to charity projects, Charity lotteries have a dual perception. On the one hand, charity lotteries are perceived as entertainment and even gambling. On the other hand, charity lotteries have a more motivating nature, encouraging an essential potential win. A volunteer movement or charitable fundraising requires high motivation in charitable, social, and environmental projects. Participation in the charity lottery does not require significant efforts from the participants. The winner gets a prize, the losers – the opportunity to participate in an essential social campaign.

In Russia, there is a practice of holding charity lotteries. An excellent example is the charity projects “Help and Win” and “Your Star”, conducted by the joint-stock company (JSC) “Russian Railways” and the public joint stock corporation (PJSC) “Aeroflot”. Passengers are invited to purchase a charity card and win a prize. The beneficiary of this lottery is the charitable foundation “Lifeline”. Currently, due to the spread of coronavirus disease (COVID-19), the sale of postcards and other non-food items on flights is suspended. The “Lifeline” foundation assesses the experience of using charity lotteries as positive; the funds received provide treatment for 70 seriously ill children a year. However, the matter of the effectiveness of the lottery as a fundraising method remains open. Currently, disposable income levels in developing countries remain low. The lottery can be attractive to consumers with its potential benefits.

The purpose of this work is to identify the prospects for using charity lotteries as a marketing tool for involving consumers and employees in charitable activities. During the study, the following objectives were set: to identify the level of consumer awareness of charity lotteries; to identify the factors that most determine the behavior of participants in charity lotteries; to define what is the position of consumers about charity lotteries, to find out whether charity lotteries create additional value for consumers.

## Methods

### Theoretical background

International studies of countries by level of charity take into account the respondent’s actions over the past month (monetary donations, volunteer activities, any help to a stranger who needed it), the number of people involved in providing charitable assistance (in %), as well as the general attitude of the people of the country to charity and volunteer activities. These studies reflect the attitude that characterizes people’s behavior to a greater extent. In our opinion, the evaluation of the charity index serves the purpose of tracking changes, helping to determine the dynamics of social achievements and comparing their activities with the best practices of other countries. However, for this study, this evaluation mechanism seems too narrow. It reflects the consequences, not the reasons for the attitude to charity. It does not consider the impact of business strategy on the involvement of consumers and employees in charity events. Therefore, the theoretical basis for our research was in socially responsible marketing tools and consumer behavior.

Based on the research review, it can be concluded that the current social image is an integral part of organizational strategies in developed countries. The authors also support the claims of many researchers that the social responsibility of an organization contributes to its competitiveness. Attention to the effectiveness of social responsibility and its impact on the organization’s competitiveness gave impetus to a detailed study of consumer behavior and their reaction to various social projects. It should be understood that consumers are significant stakeholders for the organization. Therefore, their position on social responsibility can exert pressure on the organization’s decisions.

For a perception of charitable activities to be appropriately formed, it is necessary to understand the nature of the attitude of consumers and other interested parties to philanthropic events. This research perspective remains relevant for developing countries, as CSR practices are not sustainable. A focus on CSR activities as a source of self-oriented value for consumers provides an opportunity for marketers to create differentiation and augment what is a dominant emphasis on other-oriented value in CSR research ( Peloza and Shang 2011; Azmat and Ha 2013; Anadol *et al.* 2015).

Parminder Kaur investigated consumers’ perception of the social responsibility of organizations, taking into account their desire to purchase a product, and notes that there is a positive relationship between consumer confidence, which is the result of the CSR of the company, and the intention to purchase its product ( Kaur 2013).

K. B. Bello, A. and K. Md Nor conclude the willingness of consumers to reward socially responsible companies with positive reviews. Consumers’ reaction to CSR can manifest itself in the form of trust, a higher perceived quality of service, increased customer satisfaction and loyalty, purchase intentions, and an increase in the share of purchases ( Bello *et al*. 2021).

Consumer expectations motivate marketers to incorporate social considerations into their marketing practices and communicate about those actions ( Castaldo *et al*. 2009; Chernev and Blair 2015; Golob *et al*. 2015; Chaudary *et al*. 2016). Although studies show that perception of CSR toward community has a substantial influence on consumers’ attitude when considering purchases in the distant or indefinite future, the impact of such information on purchases shortly (for example, at the time of purchase) will be quite insignificant ( Brown and Dacin 1997; Carrigan and Attalla 2001; Tian *et al.* 2011; Becker-Olsen 2014). In these situations, the consumer is more influenced by factors such as brand availability or specific functional attributes of products. If consumers believe that a company which is engaged in socially responsible activities produces outcomes which are worse for the market than firms that do not worry about social responsibilities, then information about social responsibility will not bring the desired effect ( Rivera *et al.* 2016; Wan *et al.* 2016; Schill and Godefroit 2021).

Sankar Sen and C. B. Bhattacharya note that consumers in company assessments are more sensitive to negative information about CSR than positive; moreover, all consumers react negatively to negative news, whereas positive information receives a positive reaction only from those consumers who themselves are involved in solving social problems ( Sen and Bhattacharya 2001).

The theoretical analysis allowed us to make some generalizations. Participation in charity events, including charity lotteries, has the potential to create additional value for the company and has an impact on the formation of its social image in the eyes of both consumers and employees, as well as other stakeholders ( Bhattacharya *et al.* 2009; Semeijn *et al.* 2014; Lu *et al.* 2015; Du and Sen 2016; Gürlek *et al.* 2017; Afzali and Kim 2021). However, the issue of choosing tools for involving consumers in charitable activities is poorly considered ( de Los Salmones *et al*. 2005). The World Charity Index considers only two options - financial assistance (giving money to strangers or donations) and volunteering. The potential of the charity lottery as a tool for involving consumers and employees in charitable activities remains unclear ( Hildebrand *et al.* 2017; Chen McCain *et al.* 2019; Li *et al.* 2021).

The potential of charitable events to create additional value is the subject of applied research. Analysis of research data carried out by the British Research Institute of Public Relations, Mail. Ru Corporation, and Russian Public Opinion Research Center (VTsIOM), the Charity Fund “Gift of Life (Podari Zhizn’)” allowed to identify the factors that determine the attitude to charity in general.

The British Research Institute of Public Relations conducted a study on the importance of charity for households in a sample consisting of 2,070 adults. The study revealed popular forms of participation in charity: shopping in charity shops, visiting charitable institutions, or events organized by a charitable organization. Among the factors determining the attitude to charity, gender and age were noted. An interesting result was the absence of a linear relationship between income and participation in charity.

The Mail. Ru Corporation and Russian Public Opinion Research Center (VTsIOM) conducted a study called “Attitudes towards Charity in Russia”, which examined the opinions of 1,500 respondents aged 18 to 60 living in large Russian cities. The barriers to charity identified in the study in Russia are distrust of charitable organizations (49% of respondents) and lack of money for charity (48% of respondents).

The incentive to participate in a charity event is the availability of detailed information about the action. In addition, the support of their family members and close and familiar friends is essential to users. The third incentive for respondents was the availability of various bonuses for charity. Thus, the nature of charity lotteries’ motivation helps get a positive reaction to the event.

The charity fund “Gift of Life (Podari Zhizn’)” surveyed 1,434 donors and 1,600 people who are not involved in charitable activities. The study revealed that the factors of the gender and age of respondents influenced their charity training. Education and income level also matter. Philanthropists most often are motivated in their actions. These are purposeful people who want to solve society’s problems through collective action.

Empirical studies have provided valuable information about many factors that determine people’s attitude to charity in general. There is reason to believe that the factors of awareness, the emotional coloring of the charity project, the opinions of the immediate environment, and the norms of behavior recommended by the external environment and socio-demographic characteristics are causal factors determining the reaction to the charity lottery. However, there is no direct evidence that the identified trends persist for charity lotteries.

### Research methodology

The methodology of studying people’s attitudes and their willingness to participate in the lottery is based on the principles of the Theory of Planned Behavior by Ajzen (TPB) ( Ajzen and Fisbbein 1974; Ajzen 1991). TPB defines a person’s behavior as the result of their conscious decision to act in a certain way ( Ajzen 2012, 2015; Barbera and Ajzen 2020; Bosnjak *et al*. 2020). The strengths of the TPB methodology are that it applies to various forms of behavior, takes into account a small range of influencing factors, and is easy to use. Therefore, TPB has received substantial research support ( al Jarah and Emeagwali 2017; Miller 2017; Gupta 2021).

**Figure 1.**
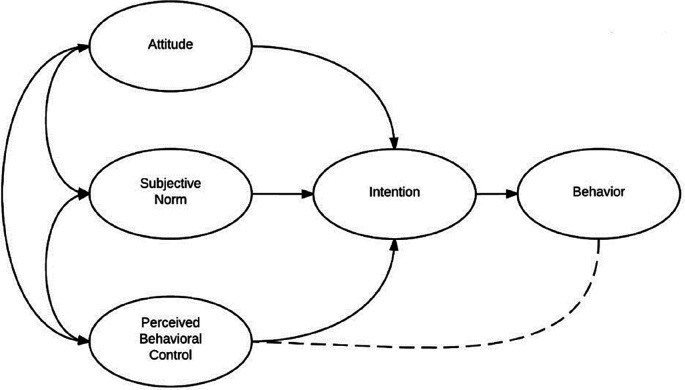
Ajzen’s theory of planned behavior (figure adapted by authors).

According to the theory of planned behavior, the choice of a person’s conscious decision is determined by three factors: attitude to behavior (Aact), subjective norms (SN) and perceived behavioral control (PBC). Together they form behavioral intention (BI) ( Ajzen 2012) ( Figure 1). The factors of the TPB model were supplemented, taking into account empirical studies. The complete list of factors is shown in Table 1. The “behavior (B)” factor was considered an influential factor.

**Table 1.** Factors influencing participation in the charity lottery (CL).

Construct (name)	Questionnaire question - observed variable	Variable
Experience	In the past, I was not aware of CL	E1
	In the past, though I was aware that CL never bought it	E2
Valuable beliefs (V)	I believe in luck	V1
	I believe in the theory of karma	V2
	I feel miracles do happen	V3
Norms of behavior (N)	People should not gamble	N1
	Buying lotteries is gambling which is against my ethics	N2
	Buying CL though for a cause is unethical as it makes people addicted to gambling	N3
Attitude towards behavior (Aact)	For me, buying CL in the next 12 months would be Good/Bad	A1
	For me, buying CL in the next 12 months would be: Foolish/Wise	A2
	For me, buying CL in the next 12 months would be: Dissatisfying/Satisfying	A3
	For me, buying CL in the next 12 months would be: Useful/Useless	A4
	For me, buying CL in the next 12 months would be: Unimportant/Important	A5
	For me, buying CL in the next 12 months would be: Desirable/Undesirable	A6
	My attitude toward CL is positive	A7
Perceived behavioral control (PBC)	I think buying CL creates a win-win situation for all	P1
	Developing and underdeveloped countries governments have limited funds to allocate to the problems faced by the nation, so floating CL is creating one more way to raise money	P2
	I think buying CL helps to try our fortune as well as raise fund for a cause	P3
	I think by buying CL, even the people who have limited means also can contribute to cause	P4
	I think a lot of times in natural disasters, people donate things that are not very useful, and the cost of collecting sometimes is more than the value things serve	P5
	I think lotteries are legal, so it helps in generating revenue for the state	P6
Subjective norms (SN)	Most people in my workplace whose opinion I value think I should not invest in CL	S1
	Most people in my workplace whose opinion I value would disapprove of my buying CL	S2
	Lot of people around me have started buying CL or intend to buy CL	S3
	My friends think I should not buy CL	S4
	My family thinks I should not buy CL	S5
	My peers think I should not buy CL	S6
	My spouse thinks I should not buy CL	S7
	I will get influenced by the person promoting CL (Celebrity Endorsement)	S8
	The reason for the lottery is not important	S9
	The Governance of the country will impact my decision to buy CL	S10
	Awareness and propaganda of CL will decide if I buy CL	S11
	I will buy CL if I can spare the money for it	S12
Intention (I)	I would buy CL in next 12 month	I1
	I will buy CL only if it happens in my state	I2
	I intend to buy CL in the next 12 months	I3
	I have decided to buy CL in the next 12 months	I4
	I am determined to buy CL in the next 12 months	I5
	For me to buy CL in the next 12 months will be	I6
	If I want to, I could buy CL in the coming 12 months	I7
	The likelihood of me buying CL is high	I8
	I expect to buy CL	I9
Behavior (B)	In the past 12 months, I have bought at least one CL	B1
	In the past 12 months, I would have bought at least one CL if I had been aware of it	B2

Two types of seven-point scales are used in the questions: a semantic differential (for Aact) and an evaluation scale with the points: 1 = Strongly Disagree, 2 = Disagree, 3 = Slightly Disagree, 4 = Neutral, 5 = Slightly Agree, 6 = Agree, and 7 = Strongly Agree.

The main hypotheses:

H1 – the stronger the attitude towards behavior (Aact), the stronger the behavioral intention (BI).

H2 – the greater the perceived behavioral control (PBC), the stronger the behavioral intention (BI).

H3 – the stronger the subjective norms (SN), the stronger the behavioral intention (BI).

H4 – the stronger the valuable belief in luck/karma, the stronger the behavioral intention (BI).

H5 – the greater the normative beliefs (NB), the stronger the behavior behavioral intention (BI).

H6 – behavioral intention (BI) is correlated with actual behavior (B).

A Structural Equation Modeling (SEM) approach was selected to analyze the cause-effect relationships among constructs used in the study.

## Results

### Attitude towards the charity lottery of respondents from Russia and the influence of socio-demographic factors on behavior

The sample was 355 people ( Barykin 2022). The respondents’ age distribution: arithmetic mean - 24.8 years, median - 21 years, total span - 49 years, standard deviation - 8.2 years. Gender distribution: male - 159 people (44.8% of respondents); female - 196 people (52.2% of respondents).

About 11% of respondents have already participated in charity lotteries. About 9% of respondents participated in charity lotteries at least once in the last year. Only 40% of respondents had an idea about the existence of such lotteries.

However, the survey showed that if the respondents knew that such an event was taking place, 48% of the respondents would participate.

### Preliminary check of influence of factors

Correlation analysis made it possible to establish a weak influence of socio-demographic factors (Age, Gender, Marital Status, Ownership of Home, Educational Qualification, Employment, Annual Income) on behavior. In further analysis, these factors were not considered when constructing the model.

The construct Experience (Exper) was insignificant and was excluded from further analysis. Variables P6 (construct PBC) and S10 (construct SN) were also removed from other research.

The results of the adjusted confirmatory factor analysis (CFA) are shown in Table 2.

**Table 2.** Regression coefficients and reliability of confirmatory factor analysis (CFA) measurement models.

Construct	Variable	Regression coefficient (non-standardized form)	Standardized regression coefficient (β)	Significance (***<0.001)	Critical ratio (CR)
Valuable beliefs (V)	V2	1.823	0.665	***	0.543
	V1	1	0.408		
	V3	1.257	0.515	***	
Norms of behavior (N)	N2	0.783	0.72	***	0.609
	N1	1	0.445		
	N3	0.59	0.577	***	
Attitude towards behavior (Aact)	A5	-1.19	-0.721	***	0.836
	A2	-0.886	-0.607	***	
	A6	1.092	0.703	***	
	A4	1.141	0.687	***	
	A3	-1.155	-0.718	***	
	A1	1	0.626		
Perceived behavioral control (PBC)	P6	1.757	0.849	***	0.835
	P2	1.008	0.551	***	
	P5	1.359	0.726	***	
	P3	1.294	0.689	***	
	P1	1	0.582		
	P4	1.165	0.641	***	
Subjective norms (SN)	S10	1.144	0.359	***	0.733
	S11	1.537	0.459	***	
	S4	1.415	0.536	***	
	S3	-1.126	-0.384	***	
	S8	1.485	0.446	***	
	S2	1.179	0.507	***	
	S1	1	0.425		
	S9	1.752	0.531	***	
	S6	1.138	0.458	***	
	S5	1.2	0.447	***	
	S7	0.929	0.358	***	
Behavior intention (BI)	BI1	0.846	0.685	***	0.880
	BI8	-0.967	-0.742	***	
	BI3	1	0.788		
	BI5	1.032	0.82	***	
	BI6	0.859	0.694	***	
	BI7	0.451	0.342	***	
	BI9	-0.905	-0.685	***	
	BI2	0.459	0.372	***	
	BI4	1.057	0.818	***	
Behavior (B)	B2	1	0.746		0.470
	B1	0.337	0.34	***	

* are used to indicate the degree of significance of the statement and characterize the magnitude of the error probability p. In the statistical software package SPSS, the value of p is denoted by Sig. (Significant). Statements with error probability p ≤ 0.001 are the most significant and are denoted by (*). Statements with a probability of error p ≤ 0.01 - are considered very significant, denoted by (**). Significant statements are denoted by *, their probability of error is p ≤ 0.05. If the error probability p is greater than 0.05, then the statement is not significant.

### Testing hypotheses about the influence of planned behavior factors

**Figure 2.**
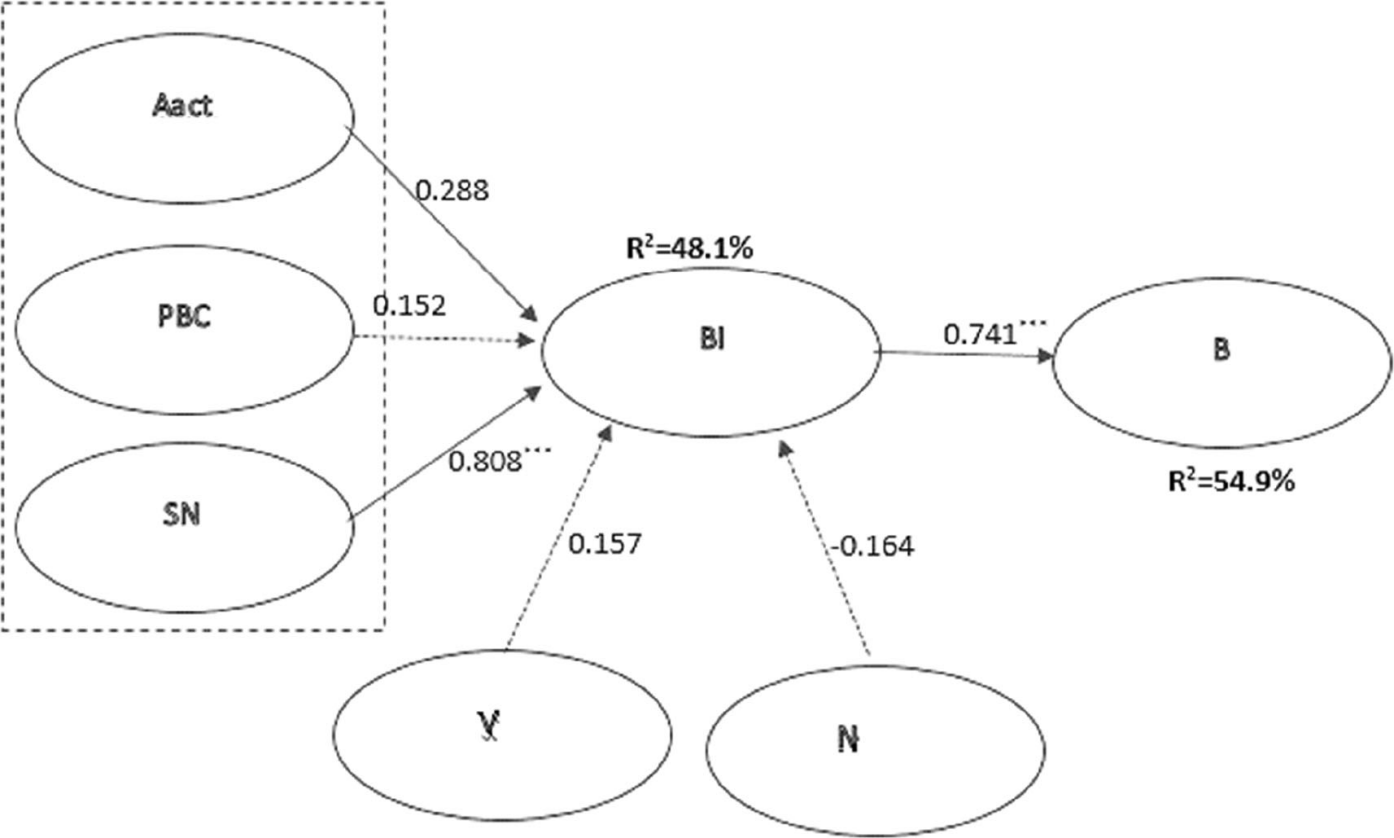
Components of the original structural model (Aact - attitude to behavior, SN - subjective norms, PBC - perceived behavioral control, BI - behavior intention, V - valuable beliefs, N - norms of behavior, B - behavior).

The results of the CFA are the basis for building a structural model (SEM), which is used to test hypotheses. The structural modeling and hypothesis testing results are presented in Figure 2 and Table 3.

**Table 3.** Hypothesis testing results (BI - behavior intention).

Hypothesis	Path	Regression coefficient	Standardized coefficient (β)	Significance	Confirmation of the hypothesis
H1	Aact→BI	0.382	0.288	0.012	Not
H2	PBC→BI	0.221	0.152	0.106	Not
H3	SN→BI	1.938	0.808	***	Yes
H4	V→BI	1.336	0.157	0.057	Not
H5	Norm→BI	-0.742	-0.164	0.048	Not
H6	BI→B	0.168	0.741	***	Yes

* are used to indicate the degree of significance of the statement and characterize the magnitude of the error probability p. In the statistical software package SPSS, the value of p is denoted by Sig. (Significant). Statements with error probability p ≤ 0.001 are the most significant and are denoted by (*). Statements with a probability of error p ≤ 0.01 - are considered very significant, denoted by (**). Significant statements are denoted by *, their probability of error is p ≤ 0.05. If the error probability p is greater than 0.05, then the statement is not significant.

## Discussion

The quality of the resulting model leaves much to be desired for predicting the behavior of Russian consumers. R ^2^ is low, i.e., some critical factors seem to be missing. It may be necessary to use more surface scales to measure variables.

Most of the research hypotheses were not confirmed. However, the influence of SN on BI was confirmed. The BI has been proven to determine the resulting behavior. However, there are quite a few factors that explain Intention. Statistical analysis of individual factors shows their connection with intention and directly with the resulting behavior. Therefore, it is not possible to separate the direct influence of these factors on behavior from the power of intentions.

It can be assumed that people are guided by more general motives for charity ( Romani *et al.* 2013; Hildebrand *et al.* 2017), while answers to questions regarding pragmatic reasons and specific values (winning, government benefits, beliefs about lotteries in general) turn out to be insignificant. It can be doubted that there is a rational internal motivation to participate in charity. Studies ( Norgaard 2006; Slote 2013; Cappelen *et al*. 2018) show that the structure of donations usually speaks in favor of the desire to avoid negative emotions, superstitious fears, and judgment, rather than in favor of the desire to help solve problems.

## Conclusions

The study showed that Russian consumers are not aware of charity lotteries. The potential of charity lotteries to engage consumers in charity remains unclear. The study has established that subjective norms define behavioral intention. Behavioral intention is correlated with actual behavior. There is reason to believe that Russian consumers make decisions about charity either impulsively (as opposed to buying clothes online) or under pressure from their environment.

The Russian specificity of charity shows the importance of trust in a charity project. Charity lottery as a form of entertainment does not build trust. The trust factor is also related to the declared informal norms of behavior and public control, which are formed by cultural traditions and the media. Russian cultural traditions favor charity donations over lotteries.

## Data availability

Figshare: Questionnaire Prospects for Using Charity Lotteries, https://doi.org/10.6084/m9.figshare.19726942.v1 ( Barykin 2022).

This project contains the following underlying data:

• Questionnaire Prospects for Using Charity Lotteries.xlsx (questionnaire responses for the study on prospects for using charity lotteries in social marketing)

Data are available under the terms of the Creative Commons Attribution 4.0 International license (CC-BY 4.0).

## Ethical approval and consent

Approval was obtained from Peter the Great St. Petersburg Polytechnic University Institutional Review Board (Approving Number F-1-Q1-2022). An informed consent form was used to obtain consent from the participants of this study. All participants voluntarily took part in this study.
