# Supplementary material for: Prospects for using charity lotteries in social marketing
Source: F1000Res. 2022 Aug 2;11:664. Originally published 2022 Jun 16. [Version 2] doi: 10.12688/f1000research.114652.2 (PMC9350437; doi:10.12688/f1000research.114652.2)
Supplement: Peer review history

# Peer review: Reviewer response for version 2

**DOI:** 10.5256/f1000research.136866.r146264

**Published:** 2022-08-03

**Authors:** Muhammad Mohsin

**Affiliations:** 1 School of Finance and Economics, Jiangsu University, Zhenjiang, China

Now the paper is ready for acceptance.

Is the work clearly and accurately presented and does it cite the current literature?

Yes

If applicable, is the statistical analysis and its interpretation appropriate?

Yes

Are all the source data underlying the results available to ensure full reproducibility?

Yes

Is the study design appropriate and is the work technically sound?

Yes

Are the conclusions drawn adequately supported by the results?

Yes

Are sufficient details of methods and analysis provided to allow replication by others?

Yes

Reviewer Expertise:

OK

I confirm that I have read this submission and believe that I have an appropriate level of expertise to confirm that it is of an acceptable scientific standard.

# Peer review: Reviewer response for version 1

**DOI:** 10.5256/f1000research.126402.r141077

**Published:** 2022-06-29

**Authors:** Muhammad Mohsin

**Affiliations:** 1 School of Finance and Economics, Jiangsu University, Zhenjiang, China

The title is interesting, and I can see merit in the paper, however I have few suggestions to improve the paper and some revisions should be made before indexing.

 The introduction section is written well and composed very well. However, a good intro presentation should be precise. The introduction needs to be more structured in terms of current citation from reputed journals.

 Although the overall language of the manuscript is good and acceptable, a few grammatical and typo issues have been observed.

 The authors partially define the amount of literature that has been carried out and where does gap persist in this area of filed. Additionally, the literature review is too short. I suggest using summary on which you reviewed the literature. Also, it would be nice to summarize the main gaps in the literature that your paper should fill, because it should be clear.

 Better to add a summarizing paragraph at the end of the introduction showing the relationship between prospects for using charity lotteries in social marketing. Overall, this section contains enough explanation however the proposed model should be compared with some popular models to measure Prospects for using charity lotteries in social marketing.

 The result and discussion sections have been presented in good structure. The result section should be compared with current and relevant citations. Again, the closing paragraph of the results can add to conclude the technical findings. Authors are suggested to improve the conclusion section since it is broadly handled and should be very concrete for describing the results followed by the policy. I would suggest a separate policy section after the conclusion. I hope my comments will help to improve the paper before publication, however, its policy implication section briefly deals with the suggestions and policy implications, which is an essential part of the description of the conclusion section.

Is the work clearly and accurately presented and does it cite the current literature?

Yes

If applicable, is the statistical analysis and its interpretation appropriate?

Yes

Are all the source data underlying the results available to ensure full reproducibility?

Yes

Is the study design appropriate and is the work technically sound?

Yes

Are the conclusions drawn adequately supported by the results?

Yes

Are sufficient details of methods and analysis provided to allow replication by others?

Yes

Reviewer Expertise:

OK

I confirm that I have read this submission and believe that I have an appropriate level of expertise to confirm that it is of an acceptable scientific standard, however I have significant reservations, as outlined above.

# Peer review: Reviewer response for version 1

**DOI:** 10.5256/f1000research.126402.r141076

**Published:** 2022-06-29

**Authors:** Yuriy Yu Shvets

**Affiliations:** 1 Department of Mathematics, Institute of Control of Science V.A. Trapeznikov RAS, Financial University under The Government of Russian Federation, Moscow, Russian Federation

Dear authors, thank you so much for giving me the opportunity to read and review your work.

 1. This work aims to identify the prospects for using charitable lotteries as a marketing tool to involve consumers and employees in charitable activities. However, in the research methodology, the authors do not specify what interpretation of involvement they use in the study of participation in a charity lottery does not require significant efforts from the participants.

 2. The authors set themselves the task of determining whether the charity lottery creates additional value for consumers. What is the potential for creating additional value for consumers in charitable lotteries themselves?

 3. The authors' conclusions did not reflect consumers’ attitudes to charitable lotteries as a gambling game. This point needs to be elaborated.

 4. In the theoretical framework, the authors discuss the issue of consumer confidence in organizations that carry out activities within the framework of corporate social responsibility. It would be appropriate to explain how this problem is reflected in the observed variables.

 5. Is there a barrier to charity in the form of a lottery if consumers have doubts about the good faith of the lottery organizer? Need a bit more explanation on this.

 6. Factors of awareness of consumer behavior by what variables were measured? Need to elaborate.

 7. What additional factors, according to the authors, should be measured additionally to improve the quality of the forecast of consumer behavior about charitable lotteries?

 8. How do the authors plan to measure the effectiveness of the lottery as a method of raising funds? This point is important to be discussed in the manuscript.

Is the work clearly and accurately presented and does it cite the current literature?

Yes

If applicable, is the statistical analysis and its interpretation appropriate?

Yes

Are all the source data underlying the results available to ensure full reproducibility?

Yes

Is the study design appropriate and is the work technically sound?

Yes

Are the conclusions drawn adequately supported by the results?

Yes

Are sufficient details of methods and analysis provided to allow replication by others?

Yes

Reviewer Expertise:

self economy

I confirm that I have read this submission and believe that I have an appropriate level of expertise to confirm that it is of an acceptable scientific standard.
